# Risk Factors for Central Lymph Node Metastases and Benefit of Prophylactic Central Lymph Node Dissection in Middle Eastern Patients With cN0 Papillary Thyroid Carcinoma

**DOI:** 10.3389/fonc.2021.819824

**Published:** 2022-01-17

**Authors:** Sandeep Kumar Parvathareddy, Abdul K. Siraj, Saeeda O. Ahmed, Felisa DeVera, Saif S. Al-Sobhi, Fouad Al-Dayel, Khawla S. Al-Kuraya

**Affiliations:** ^1^ Human Cancer Genomic Research, Research Center, King Faisal Specialist Hospital and Research Center, Riyadh, Saudi Arabia; ^2^ Department of Surgery, King Faisal Specialist Hospital and Research Center, Riyadh, Saudi Arabia; ^3^ Department of Pathology, King Faisal Specialist Hospital and Research Centre, Riyadh, Saudi Arabia

**Keywords:** head and neck cancer, papillary thyroid carcinoma, prophylactic central lymph node dissection, central lymph node metastasis, risk factors

## Abstract

**Background:**

Prophylactic central lymph node dissection (PCLND) for adult patients with papillary thyroid carcinoma (PTC) is still a matter of debate. Data on incidence, risk and benefits of PCLND in Middle Eastern patients is lacking. Therefore, we aimed to identify the incidence and predictive clinico-pathological and molecular marker of PCLND in adult patients with clinically node negative (cN0) Middle Eastern PTC.

**Methods:**

This retrospective study included 942 adult Middle Eastern patients with cN0 PTC who underwent total thyroidectomy (TT) or TT+PCLND. Clinico-pathological associations of central lymph node metastasis (CLNM) were assessed. Multivariate analysis was performed using logistic regression and Cox proportional hazards model.

**Results:**

213 patients underwent PCLND and 38.0% (81/213) had positive CLNM. Multivariate analysis demonstrated age ≤55 years (Odds Ratio (OR) = 7.38; 95% Confidence Interval (CI) = 1.59 – 34.31; p = 0.0108), tumor bilaterality (OR = 3.01; 95% CI = 1.01 – 9.21; p = 0.0483), lymphovascular invasion (OR = 2.92; 95% CI = 1.18 – 7.23; p = 0.0206) and *BRAF* mutation (OR = 3.24; 95% CI = 1.41 – 7.49; p = 0.0058) were independent predictors of CLNM in adult PTC. Furthermore, patients who underwent PCLND showed significant association with improved recurrence-free survival (RFS; p = 0.0379). Multivariate analysis demonstrated that PCLND was an independent predictor of improved recurrence-free survival.

**Conclusions:**

cN0 Middle Eastern PTC patients treated with PCLND showed a significantly better prognosis. PCLND was effective in improving RFS in Middle Eastern PTC patients and should be encouraged for patients with potential risk factors for CLNM.

## Introduction

During the past two decades, the incidence of thyroid cancer has continually increased (1, 2). Papillary thyroid carcinoma (PTC) accounts for the majority of thyroid cancers (3). PTC has become the second most prevalent carcinoma among women in Saudi Arabia (4). Most PTC patients have an excellent prognosis (5). However, despite this favorable outcome, PTC is prone to spread to the central lymph node (CLN). In fact, 20 – 40% of PTC patients have been reported to have CLN metastasis (CLNM) even in clinically node negative patients (6–9).

The incidence of occult node metastasis is high and reaches up to 90% according to several reports (10–13). Furthermore, CLNM have been reported to increase the risk of recurrence and disease-specific mortality (14–16). Recurrence in PTC remains a big challenge and affects the optimal therapeutic strategy for PTC patients. Although central lymph node dissection (CLND) is recommended for patients who are suspected of CLNM in preoperative evaluation, there are still no clear guidelines for CLND for patients with clinically node negative (cN0) PTC.

In surgical treatments, prophylactic CLND (PCLND) for patients with PTC is still a controversial issue. Some studies have shown that PCLND could be beneficial in terms of reducing locoregional recurrence and thus improving the disease-free survival. In addition, PCLND could also help in more precise staging of PTC, since nodal positivity could convert patients from cN0 to pN1a, leading many patients over 55 years of age from AJCC stage I to AJCC stage III, which in turn changes the therapeutic choices (17–20). However, other studies consider the risk of postoperative complications (such as laryngeal nerve injury and hypoparathyroidism) following PCLND to outweigh the potential benefits, and thus do not recommend the routine use of PCLND in PTC (21–24). Although the American Thyroid Association (ATA) Guidelines suggest PCLND be considered, especially for PTC patients who present with advanced tumors (T3 and T4) (25), the endocrine community is still debating the guidelines suggestion data.

However, the benefits of PCLND in Middle Eastern patients with cN0 PTC has not been fully explored. Therefore, we conducted this retrospective study on a large cohort of Middle Eastern PTC to identify both the incidence and the predictive parameters of CLNM in cN0 PTC patients. We then assessed, for the first time, the benefit of PCLND with clinically node negative PTC in reducing the incidence of recurrent disease and improving patient outcome. Data from this study may give additional guidance on the surgical and therapeutic decision for PTC patients from Middle Eastern ethnicity.

## Materials and Methods

### Patient Selection

Nine hundred and forty-two PTC patients diagnosed between 1988 and 2018 at King Faisal Specialist Hospital and Research Centre (Riyadh, Saudi Arabia) were included in the study. Inclusion criteria were: 1) PTC confirmed by fine needle aspiration cytology; 2) no evidence of lymph node metastasis at either clinical examination or on ultrasound; and 3) more than 18 years of age. The Institutional Review Board of the hospital approved this study and the Research Advisory Council (RAC) provided waiver of consent under project RAC # 221 1168 and # 2110 031.

### Clinico-Pathological Data

Patients underwent total thyroidectomy with (n = 213) or without (n = 729) PCLND. PCLND was performed in patients with clinically uninvolved central neck lymph nodes (cN0) who had advanced primary tumors (T3 or T4) or clinically involved lateral neck nodes (cN1b), or if the information could be used to plan further steps in therapy, in accordance with the 2015 ATA guidelines (25). Baseline clinico-pathological data were collected from case records and have been summarized in **Table 1**. Clinico-pathological data included patients’ age at diagnosis, gender, histologic subtype, tumor laterality, focality, extrathyroidal extension, lymphovasular invasion, tumor size, central lymph node status, stage and tumor recurrence. Staging of PTC was performed using the eighth edition of AJCC staging system. Only structural recurrence (local, regional or distant) was considered for analysis. Recurrence was defined as any newly detected tumor (local or distant) or metastatic regional lymph node (LN) based on ultrasound and/or imaging studies in patients who had been previously free of disease following initial treatment. Risk categories were defined based on 2015 ATA guidelines (25).

**Table 1:** Clinico-pathological and molecular characteristics of adult papillary thyroid carcinoma.

	No.	%
Total	942	
Age in years, Median (range)	40.0 (18.1 – 89.0)
Age (years)		
<55	751	79.7
≥55	191	20.3
Gender		
Female	724	76.9
Male	218	23.1
Histologic subtype		
Classical variant	565	60.0
Follicular variant	214	22.7
Tall cell variant	91	9.7
Other variants	72	7.6
Tumor laterality		
Unilateral	686	72.8
Bilateral	256	27.2
Multifocality		
Yes	429	45.5
No	513	54.5
Extrathyroidal extension		
Present	322	34.2
Absent	620	65.8
Lymphovascular invasion		
Present	198	21.0
Absent	744	79.0
Microcarcinoma		
Yes	134	14.2
No	808	85.8
pT		
T1	392	41.6
T2	311	33.0
T3	183	19.4
T4	56	5.9
pM		
M0	911	96.7
M1	31	3.3
TNM Stage		
I	788	86.0
II	89	9.7
III	11	1.2
IV-A	12	1.3
IV-B	16	1.8
*BRAF* mutation		
Present	481	51.1
Absent	438	46.5
Unknown	23	2.4
*TERT* mutation		
Present	118	12.5
Absent	749	79.5
Unknown	75	8.0
Recurrence		
Yes	125	13.3
No	817	86.7
Type of surgery		
Thyroidectomy	729	77.4
Thyroidectomy + PCLND	213	22.6
Radioactive Iodine therapy		
Yes	754	80.0
No	188	20.0
ATA risk category		
Low	233	24.7
Intermediate	327	34.7
High	382	40.6
Total follow-up duration		
Median (range), in years	9.1 (1.0 – 30.1)

### *BRAF* and *TERT* Mutation Analysis

*BRAF* and *TERT* mutation data was assessed in our laboratory by Sanger sequencing and has been published by us previously (26, 27).

### Follow-Up and Study Endpoint

Patients were regularly followed by both physical examinations and imaging studies to identify tumor recurrence. The median follow-up was 9.1 years (range 1.0 – 30.1 years). Recurrence-free survival (RFS) was defined as the time (in months) from date of initial surgery to the occurrence of any tumor recurrence (local, regional or distant). In case of no recurrence, date of last follow-up was the study endpoint for RFS.

### Statistical Analysis

The associations between clinico-pathological variables was performed using contingency table analysis and Chi square tests. Mantel-Cox log-rank test was used to evaluate recurrence-free survival. Survival curves were generated using the Kaplan-Meier method. Logistic regression and Cox proportional hazards model were used for multivariate analysis. Clinico-pathological variables adjusted in multivariate analysis were age, sex, histology, laterality, focality, extrathyroidal extension, lymphovascular invasion, pT, pN, stage, *BRAF* mutation and *TERT* mutation. Two-sided tests were used for statistical analyses with a limit of significance defined as p value < 0.05. All data analyses were performed using the JMP14.0 (SAS Institute, Inc., Cary, NC) software package.

## Results

### Patient and Tumor Characteristics

Median age of the study cohort was 40.0 years (range = 18.1 – 89.0 years), with a male: female ratio of 1:3.3. Classical variant PTC was the predominant histologic subtype, accounting for 60.0% (565/942) of all cases, followed by follicular variant (22.7%; 214/942) and tall cell variant (9.7%; 91/942). Extrathyroidal extension was noted in 34.2% (322/942) of cases and lymphovascular invasion in 21.0% (198/942). 45.5% (429/942) of PTCs were multifocal and 27.2% (256/942) were bilateral. 80.0% (754/942) of PTC patients in our cohort received radioactive iodine (RAI) therapy. Tumor recurrence was noted in 13.3% (125/942) of the entire cohort (**Table 1**). However, among the 213 patients who underwent PCLND, tumor recurrence was seen in only 8.5% (18/213) (**Table 2**).

**Table 2:** Clinico-pathological characteristics stratified by central lymph node metastasis (CLNM) status in adult cN0 PTC patients who underwent prophylactic central lymph node dissection.

	Total		CLNM present		CLNM absent		p value
	No.	%	No.	%	No.	%	
Total	213		81	38.0	132	62.0	
Age at surgery (years)							
<55	171	80.3	72	88.9	99	75.0	0.0106
≥55	42	19.7	9	11.1	33	25.0	
Gender							
Female	160	75.1	67	82.7	93	70.4	0.0407
Male	53	24.9	14	17.3	39	29.6	
Histologic subtype							
Classical variant	120	56.3	53	65.4	67	50.8	0.0001
Follicular variant	45	21.1	5	6.2	40	30.3	
Tall cell variant	34	16.0	17	21.0	17	12.9	
Other variants	14	6.6	6	7.4	8	6.0	
Tumor laterality							
Unilateral	147	69.0	49	60.5	98	74.2	0.0364
Bilateral	66	31.0	32	39.5	34	25.8	
Extrathyroidal extension							
Present	84	39.4	38	46.9	46	34.9	0.0811
Absent	129	60.6	43	53.1	86	66.5	
Multifocality							
Yes	106	49.8	50	61.7	56	42.4	0.0060
No	107	50.2	31	38.3	76	57.6	
Lymphovascular invasion							
Present	58	27.2	29	35.8	29	22.0	0.0290
Absent	155	72.8	52	64.2	103	78.0	
Microcarcinoma							
Yes	22	10.4	9	11.1	13	9.9	0.7697
No	191	89.6	72	88.9	119	90.1	
pT							
T1	46	21.6	29	35.8	17	12.9	<0.0001
T2	38	17.9	29	35.8	9	6.8	
T3	107	50.2	19	23.5	88	66.7	
T4	22	10.3	4	4.9	18	13.6	
pM							
M0	209	98.1	80	98.8	129	97.7	0.5768
M1	4	1.9	1	1.2	3	2.3	
TNM Stage							
I	174	82.1	72	88.9	102	77.9	0.1883
II	26	12.2	6	7.4	20	15.3	
III	5	2.4	2	2.5	3	2.3	
IV-A	5	2.4	1	1.2	4	3.0	
IV-B	2	0.9	0	0.0	2	1.5	
*BRAF* mutation							
Present	116	56.3	57	74.0	59	45.7	<0.0001
Absent	90	43.7	20	26.0	70	54.3	
*TERT* mutation							
Present	25	12.8	8	10.5	17	14.2	0.4517
Absent	171	87.2	68	89.5	103	85.8	
Recurrence							
Yes	18	8.5	10	12.4	8	6.1	0.1153
No	195	91.5	71	87.6	124	93.9	
ATA risk category							
Low	42	19.7	10	12.4	32	24.2	0.0828
Intermediate	78	36.6	34	42.0	44	33.3	
High	93	43.7	37	45.7	56	42.4	

### Clinico-Pathological Associations of CLNM

Of the 213 PTC patients that underwent PCLND, CLNM was noted in 38.0% (81/213) of cases. Presence of CLNM in these patients was significantly associated with adverse clinico-pathological risk factors, such as tall cell variant (p = 0.0001), bilateral tumors (p = 0.0364), multifocality (p = 0.0060), lymphovascular invasion (p = 0.0290) and *BRAF* mutation (p < 0.0001). Interestingly, we also found that CLNM was associated with younger age (< 55 years; p = 0.0106) and female sex (p = 0.0407). CLNM was also found to be more frequent in PTCs with extrathyroidal extension, although the difference was not statistically significant (p = 0.0811) (**Table 2**).

### Risk Factors Predicting CLNM

We analyzed the clinico-pathological risk factors that could independently predict CLNM in patients undergoing PCLND. Using multivariate logistic regression analysis, we found age < 55 years (Odds ratio (OR) = 7.38; 95% Confidence interval (CI) = 1.59 – 34.31; p = 0.0108), bilateral tumors (OR = 3.01; 95% CI = 1.01 – 9.21; p = 0.0483), lymphovascular invasion (OR = 2.92; 95% CI = 1.18 – 7.23; p = 0.0206) and *BRAF* mutation (OR = 3.24; 95% CI = 1.41 – 7.49; p = 0.0058) to be independent predictors for CLNM (**Table 3**).

**Table 3:** Independent predictors of central lymph node metastasis by multivariate logistic regression analysis.

Clinico-pathological variables	Central Lymph node metastasis		
	Odds ratio	95% Confidence Interval	p value
**Age**			
< 55 years (vs. ≥ 55 years)	7.38	1.59 – 34.31	0.0108
**Sex**			
Male (vs. Female)	0.75	0.27 – 2.06	0.5743
**Histology**			
Tall cell variant (vs. other variants)	1.09	0.38 – 3.16	0.8692
**Tumor laterality**			
Bilateral (vs. Unilateral)	3.01	1.01 – 9.21	0.0483
**Tumor focality**			
Multifocal (vs. Unifocal)	0.97	0.34 – 2.77	0.9482
**Extrathyroidal extension**			
Present (vs. Absent)	1.44	0.60 – 3.50	0.4131
**Lymphovascular invasion**			
Present (vs. Absent)	2.92	1.18 – 7.23	0.0206
**pT**			
T3-4 (vs. T1-2)	0.06	0.02 – 0.15	<0.0001
**Stage**			
III-IV (vs. I-II)	1.99	0.22 – 17.99	0.5407
***BRAF* mutation**			
Present (vs. Absent)	3.24	1.41 – 7.49	0.0058
***TERT* mutation**			
Present (vs. Absent)	2.20	0.45 – 10.71	0.3285

We next to sought to determine if the combination of BRAF mutation with any of the other independent clinical parameters leads to better prediction of CLNM. We found that BRAF + age < 55 years had a better predictive value compared to age alone (OR – 3.91 vs. 2.67). Similarly, BRAF + bilateral tumors had a better predictive value compared to bilateral tumors alone (OR – 2.52 vs. 1.88) and BRAF + lymphovascular invasion had a better predictive value compared to lymphovascular invasion alone (OR – 3.97 vs 1.98) (**Table 4**).

**Table 4:** Combination of *BRAF* mutation and other risk factors as predictors of central lymph node metastasis.

Clinico-pathological variables	Central Lymph node metastasis		
	Odds ratio	95% Confidence Interval	p value
***BRAF* mutation + Age < 55 years**	3.91	2.18 – 7.01	<0.0001
**Age <55 years**	2.67	1.20 – 5.92	0.0159
***BRAF* mutation + Bilateral tumors**	2.52	1.24 – 5.11	0.0103
**Bilateral tumors**	1.88	1.04 – 3.40	0.0363
***BRAF* mutation + Lymphovascular invasion**	3.97	1.85 – 8.52	0.0004
**Lymphovascular invasion**	1.98	1.07 – 3.66	0.0289

### Prognostic Impact of PCLND

**Figure 1:**
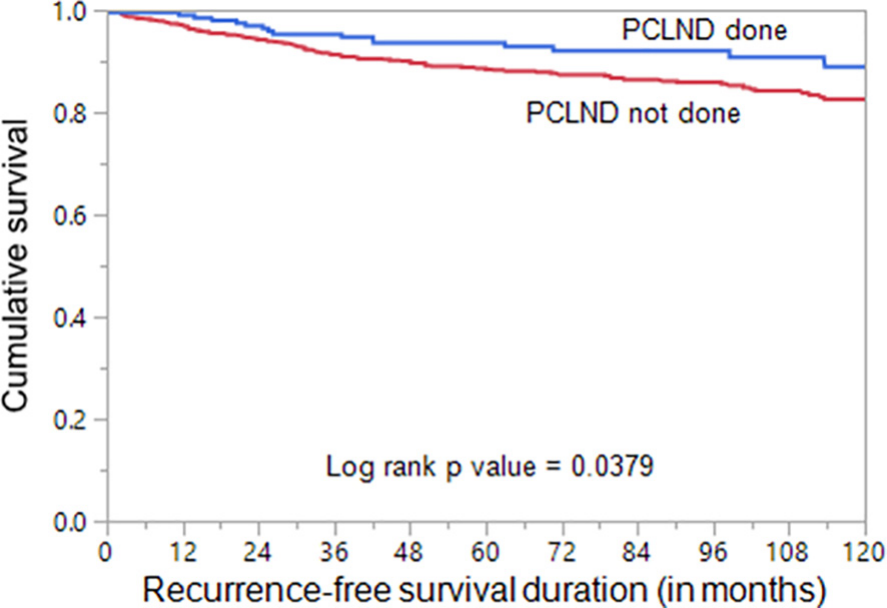
Recurrence-free survival analysis of prophylactic central lymph node dissection (PCLND) in clinically node negative (cN0) PTC. Kaplan Meier survival plot showing statistically significant improved recurrence-free survival in patients undergoing PCLND compared to those that did not (p = 0.0379).

We found that cN0 patients undergoing PCLND had a significantly reduced recurrence rate compared to patients who did not undergo PCLND (8.5% vs. 14.7%; p = 0.0138). To determine whether performing PCLND truly had an impact on recurrence, we analyzed the recurrence-free survival using Kaplan Meier curves and multivariate Cox proportional hazards model. Kaplan Meier curves showed that patients undergoing PCLND had a significantly better recurrence-free survival compared to those who did not (p = 0.0379; **Figure 1**). On multivariate analysis, PCLND was found to be an independent predictor of improved recurrence-free survival (Hazard ratio = 0.36; 95% confidence interval = 0.20 – 0.63; p = 0.0002) (**Table 5**).

**Table 5:** Independent predictors of recurrence-free survival in papillary thyroid carcinoma.

Clinico-pathological variables	Recurrence-free survival		
	Hazard ratio	95% Confidence Interval	P value
Age			
≥55 years (vs. <55 years)	1.97	1.13 – 3.31	0.0166
Sex			
Male (vs. Female)	1.99	1.33 – 2.97	0.0011
Histology			
Tall cell variant (vs. other variants)	0.66	0.32 – 1.22	0.1921
Tumor laterality			
Bilateral (vs. Unilateral)	1.90	1.04 – 3.71	0.0369
Tumor focality			
Multifocal (vs. Unifocal)	0.97	0.49 – 1.76	0.9132
Extrathyroidal extension			
Present (vs. Absent)	1.53	0.95 – 2.48	0.0801
Lymphovascular invasion			
Present (vs. Absent)	0.81	0.48 – 1.30	0.3816
pT			
T3-4 (vs. T1-2)	1.83	1.12 – 2.99	0.0172
pN			
N1 (vs. N0)	1.77	1.37 – 2.73	0.0119
Stage			
III-IV (vs. I-II)	1.21	0.59 – 2.48	0.5945
*BRAF* mutation			
Present (vs. Absent)	0.77	0.50 – 1.81	0.2264
*TERT* mutation			
Present (vs. Absent)	3.98	2.45 – 6.39	<0.0001
PCLND			
Done (vs. not done)	0.36	0.20 – 0.63	0.0002

## Discussion

PTC is common in Middle Eastern population with favorable long term prognosis (4, 28, 29). The fundamental therapy of PTC is surgery. When macroscopic nodal disease is present, either detected on imaging preoperatively or identified intraoperatively, lymph node dissection is the ideal management (25, 30, 31) due to the heightened risk of recurrence. However, the management of microscopic lymph node involvement, which is present in subset of patients, is unclear (32–34). The indications for prophylactic central neck dissection in patients with cN0 remains a subject of debate in PTC surgical management (12, 20, 35). The prevalence and the predictors of central nodal metastasis of cN0 Middle Eastern PTC have not been explored. Therefore, we aimed in this work to evaluate the incidence and the predictive factors of CNLM of cN0 in Middle Eastern PTC and focused our attention on the effect of PCNLD in patient’s prognosis.

In this study, we included 942 adult PTC patients with clinically uninvolved lymph nodes (cN0). PCLND was performed in 22.6% (213/942) patients while thyroidectomy alone was performed in 77.4% (729/942) of the PTC patients. Among those who underwent PCLND, 38% (81/213) of PTC patients were found to have CLNM by histopathology post-operative result, which implies that occult metastatic lymph nodes would have remained undetected if PCLND was not performed. This is of great clinical value for PTC patients from Middle Eastern ethnicity where recurrence rate is relatively high (36–38). Even in this cohort, which included only cN0 PTCs, recurrence rate was 13%. Presence of occult LNM and incomplete CLND could be attributed to this high recurrence rate. The recurrence rate dropped to 8.5% in patients who underwent PCNLD. Thus, we attempted to estimate the risk factors of CLNM and identify accurate predictors of CNLM in this population.

The age factor was found to have great impact on lymph node metastasis in PTC. Previous study has shown that CLNM is significantly higher in younger (<45 years) patients (39). Another recent study conducted on Chinese PTC cohort suggested patients ≤ 35 years old are more vulnerable to CLNM (8). In our study, we used the age cut off of 55 years according to the recent AJCC criteria, and found that age was predictive of occult CLNM, with patients aged < 55 were more likely to have occult CLNM (p = 0.0106). Moreover, we found that age was an independent risk factor for CLNM in cN0 adult patients with PTC (OR = 7.38; 95% CI = 1.59 – 34.31; p = 0.0108). Gender was previously reported to influence the LN metastasis, with male patients considered to have higher risk for CLNM than female patients (8, 9, 40). The risk differences might possibly be explained by the different levels of hormone in females and males. However, our study showed opposite result where CLNM is higher in female PTC patients. However, gender was not an independent predictive factor for CLNM in multivariate analysis.

Among the clinico-pathological markers that were assessed before and during surgery, we found that tumor bilaterality and lymphovascular invasion were independent predictors for CLNM in cN0 PTC. In agreement with our finding, lymphovascular invasion has been reported to adversely influence PTC biological behavior with regards to CLNM and survival (41–43). While some previous reports have documented the association between tumor bilaterality and LNM as well as local-regional recurrence (44–46), others did not consider bilateral PTC as an independent prognostic factor (47).

Advances in translational medicine have highlighted the prognostic impact of novel genetic alterations in PTC. We sought to investigate the usefulness of these molecular markers (*TERT* and *BRAF* mutations) as predictors for CLNM, since alterations of both genes are common in PTC and known to be associated with PTC aggressiveness, recurrence and metastasis (27, 48–50). In our study, we could not find association between *TERT* mutations and CLNM in cN0 PTC. However, we found *BRAF* mutation to be an independent predictor of occult CLNM (OR = 3.24; 95% CI = 1.41 – 7.49; p = 0.0058). Interestingly, we found that combining BRAF mutation with any of the other independent clinical parameters identified in this study, could increase the prediction power for CLNM. This could help in more accurate stratification of PTC patient into distinct risk groups and help in better therapeutic approach and outcome.

Considering the ATA risk stratification for structural recurrence, patients with CLNM were more often classified in the intermediate and high risk group. This is consistent with Medas et al. (17), where CLNM was significantly associated with ATA intermediate risk. This might be attributed to the PCLND which usually allows better assessment of LN and more accurate staging. Importantly, patients who underwent PCLND showed significantly better recurrence free survival. Moreover, multivariate analysis showed that PCLND was an independent predictor of better RFS regardless of other clinico-pathological variables. This is of high clinical importance since it shows the benefit from aggressive surgery in modifying the outcome of the disease and significantly improved the recurrence free survival in Middle Eastern PTC patients, where recurrence rate is relatively high. In concordance with our findings, two previous meta-analyses conducted on PTC patients from different ethnicity have shown that PCLND is beneficial in reducing risk of recurrence (51, 52).

There are several limitations of this study. Firstly, it is a retrospective single institute study, so selection bias should be considered. Secondly, this study lacks information about post-surgical risk of hypothyroidism and laryngeal nerve injury. Thirdly, all the patients included were from Middle Eastern ethnicity, so applying conclusions to other races should be done with caution.

In summary, CLNM occurred in 38.0% of cN0 adult PTC patients undergoing PCLND; multivariate logistic regression analysis revealed age, tumor bilaterality, lymphovascular invasion and *BRAF* mutations as independent risk factors for CLNM in adult PTC patients. In addition, our work succeeded in demonstrating better prognosis in patients who underwent PCLND and performing PCLND was an independent marker to improve the RFS. We believe that, in adult Middle Eastern patients with these risk factors, PCLND should be performed in order to reduce the risk of CLNM and to improve patients’ outcome.

## Data Availability Statement

The original contributions presented in the study are included in the article/supplementary material. Further inquiries can be directed to the corresponding author.

## Ethics Statement

The studies involving human participants were reviewed and approved by Research Advisory Council, King Faisal Specialist Hospital and Research Centre. Written informed consent for participation was not required for this study in accordance with the national legislation and the institutional requirements.

## Author Contributions

SP and AS analyzed the clinical data, designed and wrote the manuscript. SP performed statistical analysis. FD and SA performed clinical data abstraction. SA-S and FA-D contributed samples and analyzed clinical data. KA-K designed, implemented the study, wrote and critically reviewed the manuscript. This is to confirm that all authors read and approved the final manuscript.

## Conflict of Interest

The authors declare that the research was conducted in the absence of any commercial or financial relationships that could be construed as a potential conflict of interest.

## Publisher’s Note

All claims expressed in this article are solely those of the authors and do not necessarily represent those of their affiliated organizations, or those of the publisher, the editors and the reviewers. Any product that may be evaluated in this article, or claim that may be made by its manufacturer, is not guaranteed or endorsed by the publisher.
